# Determinants of underweight among children aged 0–23 months in Tanzania

**DOI:** 10.1002/fsn3.2748

**Published:** 2022-01-18

**Authors:** Cypriana Cyprian Moshi, Penina Joseph Sebastian, Devotha Gabriel Mushumbusi, Kaunara Ally Azizi, Wessy Pirbhai Meghji, Malimi Emmanuel Kitunda, Ladislaus Manaku Kasankala

**Affiliations:** ^1^ Department of Food Science and Nutrition Tanzania Food and Nutrition Center Dar es Salaam Tanzania

**Keywords:** BMI, determinants, Tanzania, under five, underweight

## Abstract

Underweight is the most dependable growth indicator for overall child growth. Tanzania has the highest rate of underweight children in East Africa, with 1.27 million children under the age of five suffering from the condition. This study aimed to determine factors that influence underweight in Tanzanian children aged 0–23 months. We used data from the Tanzania Demographic and Health Survey (TDHS) 2015–2016 to conduct secondary analysis on a sample of 4,327 children aged 0–23 months. Descriptive and inferential statistics such as frequency, chi‐square, binary, and multivariate logistic regression were performed using the Statistical Package of Social Science (SPSS version 25). Statistical significance was defined as a *p*‐value of less than 0.05. Multivariate analysis found risk factors for underweight children were as follows**:** child's gender, age, birth weight, mothers' BMI, level of education, and type of toilet facility used by the households. Females had a significantly lower risk of being underweight (AOR = 0.62, 95% CI = 0.48–0.81, *p* < .05) compared with male children. The odds of being underweight increase with low birth weight (AOR = 2.92, 95% CI = 1.92–4.43, *p* < .05), low mother's BMI (AOR = 2.48, 95% CI = 1.34–4.58, *p* < .05), and low educational level (AOR = 1.78, 95% CI = 1.23–2.58, *p* < .05). Nutrition interventions such as growth monitoring, nutrition counseling, and nutrition education for parents/caregivers are critical to ensuring proper weight gain for all children under 2 years of age.

## Graphical abstract

Underweight is recognized as an important indicator of health and nutritional status in the population. Determinants of underweight among children aged 0–23 months in Tanzania are not well documented. Secondary data analysis on a sample of 4327 children aged 0–23 months was done to determine factors that influence underweight in Tanzanian children.

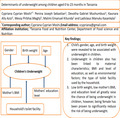

**Key Message**

- Any form of malnutrition in children aged from 0 to 23 months is an alarming threat since its consequences may persist even in adults and they cannot be reversed.
- Gender, age, birth weight, mothers' BMI and education level, and type of toilet facility available to the household were all risk factors for underweight in children under the age of 2 years.
- Nutrition interventions such as growth monitoring, nutrition counseling, and nutrition education for parents/caregivers are critical to ensuring that all children gain the appropriate amount of weight.

## 1 INTRODUCTION

Underweight is recognized as an important indicator of health and nutritional status in the population, and it is manifested when a child becomes either thin or short for her/his age (UNICEF et al., 2020). Despite a global drop in underweight children from 25% in 1990 to 15% in 2015, the decline was not uniformly spread across the globe, with about 90% of underweight children found in Southeast Asia and Sub‐Saharan Africa (WHO, 2015) and (United Nation, 2015). In Tanzania, according to the 2018 National Nutrition Survey, 2,562 children under the age of five were underweight, of which 40% were under two (United Republic of Tanzania, 2019).

Underweight is associated with multiple factors like occupation and education level of caregivers, low birth weight, age of children, birth order, sex, and decision making (Akombi et al., 2017; Balogun & Yakubu, 2015; Novignon et al., 2015; Sunguya et al., 2019). Other studies suggest a significant association between being underweight and consanguinity, late initiation of breastfeeding, dietary diversity, inadequate food intake, frequency of feeding, and poor complimentary food (Hasnain & Hashmi, 2009; Asfaw et al., 2015 and Idris et al., 2012). Also, the mother's BMI, gender of family's head, family size, and maternal age correlate with being underweight for a child (Nyaruhucha et al., 2006; Nolla et al., 2014 and Sapkota, 2009). Similarly, poor access to clean drinking water, type of resident (rural or urban), presence and use of toilets, hand washing habits, and episodes of diarrhea are associated with underweight in children under the age of five (Demilew & Abie, 2017; Yisak et al., 2015).

While there has been a reduction of underweight prevalence to 14.6% in Tanzania, the prevalence is still an alarming threat, affecting an equivalent estimate of 1.27 million under‐five children every year (United Republic of Tanzania, 2019). The World Health Organization (WHO) categorizes the severity of underweight prevalence within the range of 10%–19% among children under 5 years of age as medium (WHO, 1997). It is worth noting that the mortality risk has no exception either to mildly underweight or severely underweight children; both are at greater risk (Rodriguez‐leyva & Pierce, 2021). The country is still experiencing a mortality rate of 38.4% attributed to unsafe water, unsafe sanitation, and lack of hygiene. In addressing the consequences of malnutrition, the country implements interventions largely focused on addressing the immediate causes, underlying causes, and basic causes of under nutrition. These are well stipulated in the National Multi‐sectorial Nutrition Action Plan (NMNAP) (United Republic of Tanzania, 2019). A combination of nutrition interventions with environmental sanitation strategies like waste disposal, access to clean water, and use of clean household fuels is guided by evidence to policy to action for 5 years.

In Tanzania, limited research evidence exists which explores the determinants of underweight for children aged between 0 and 23 months. Most of these studies targeted specific regions and all children under 5 years of age without considering the critical child age from 0 to 23 months. Any form of malnutrition in this age group is an alarming threat since its consequences may persist even in adults and they cannot be reversed. Therefore, this study aimed to find out what factors influence underweight in Tanzanian children aged 0–23 months. The findings of this study will help nutrition stakeholders plan and create nutrition programs that will reduce underweight in this age group, ensuring their lives and the economic advancement of future generations. Also, contribute to achieving sustainable development goal number 2, which aims to eliminate all types of malnutrition by 2030.

## 2 METHODOLOGY

The study examined the secondary data obtained from the Tanzania Demographic and Health Survey (TDHS) 2015–2016 dataset. The survey was a cross‐sectional conducted by the National Bureau of Statistics in collaboration with other government partners. The survey used a multi‐stage sampling design composed of 608 clusters, and from each cluster, 22 households were systematically sampled. The survey involved 10,233 children aged 0–59 months and their mothers or caregivers. The present study was restricted to only 4,327 children aged 0–23 months and their anthropometric data on weights. The weight measurements of children were collected by trained enumerators using a SECA Uni scale electronic with a precision of 100 g. The weighing was done on a naked child as recommended for anthropometric methods, and if a mother/caregiver refused to remove clothes for her child, the enumerators recorded the weight as per instruction. A younger child who was not able to stand on the scale was measured on his/her caregiver's hand using the mother‐to‐baby function of the scale. The obtained weight was entered into the WHO standard software to give underweight, that is, weight for age Z‐scores. The child was considered to be underweight if his/her weight for age Z‐score (WAZ) was below −2 SD, grouped as moderate underweight when the weight for age Z‐score ranged from −2 to −3 SD, and severe underweight if the weight for age Z‐score ranged below −3 SD. The calculated weight for the age Z‐score (WAZ) was based on 2006 WHO standards.

To determine the risk factors, the underweight was analyzed against independent variables at the individual, mother, and household level. Individual‐level were child age, sex, birth weight, vitamin A supplements, if given a worm infestation drug (deworming), and an episode of diarrhea 2 weeks before the survey. The mothers' age, body mass index, and education level were analyzed at the mother's level, while the type of residence (rural or urban), type of toilet (improved or unimproved), sources of water (improved or unimproved), and time spent on getting water were analyzed as household factors. Furthermore, child feeding practices like the extent of exclusive breastfeeding, continuing breastfeeding for 1 or 2 years, minimum dietary diversity, and minimum meal frequency were analyzed. The households were assessed against an improved toilet, which was supposed to be a toilet with a flush or pour‐flush toilet connected to a piped sewer system, septic tank, pit latrine, ventilated improved pit (VIP) latrine, and pit latrine with a slab or composting toilet.

The independent variables at the individual level were assessed through self‐reports of the child's mother during interviews. The children's receiving of vitamin A and deworming drugs was assessed by recalling the children's mothers at 6 months. Assessment of the onset of diarrhea by recalling the child's mother for 14 days. Assessment of child feeding practices including breastfeeding was done through 24‐h memories of the mother. In addition, sociodemographic characteristics like age and education level were mainly self‐reported by the mother while environmental factors like type of water source and toilet facility were observed by the interviewer during the interview.

### 2.1 Data analysis

During data analysis, the Statistical Package for Social Science (SPSS) version 25 was used. Initially, the recorded data were cleaned, transformed, and recorded in the recommended format as per this study. The prevalence was calculated for all categorical data. The outcome variable for this study was underweight, which was recorded as binomial data with “1” coded as normal weight and “0” as underweight. The association between dependent and selected determinant factors was done using Chi‐square. Bivariate and multivariate logistic regression was done to determine the odds of determinant variables showing significant association with dependent variables. The backward elimination process was selected during multivariate analysis to assess factors that were significantly associated with the study outcome using a 5% significance level. To avoid or minimize statistical error, multicollinearity was tested. The odds ratios with 95% CIs were calculated in order to assess the adjusted risk of independent variables, and those with *p* < .05 were retained in the final model.). Statistical significance was defined as a *p*‐value of less than 0.05.

## 3 RESULTS

### 3.1 Social demographic characteristics of study's participants

The age group was equally represented, with a slight increase observed for 12–17 months (Table 1). About 17.6% of children experienced diarrhea in 2 weeks before the survey. The majority of children (93.4%) were born with an average or above‐average weight. Over 50% of the children had not received vitamin A supplements and deworming tablets for 6 months before the survey. The minority of mothers (15.1%) who participated in the study were aged 35–49 years, and 7.3% had a BMI less than 18.5 which is considered underweight. Furthermore, over half of the mothers (63.8%) who participated in this study attained primary education. Also, the majority of study participants, 72% and 74.2%, respectively, live in rural areas and came from households that had unimproved toilet facilities.

**TABLE 1:** Description of children, mothers, and household characteristics

Variables	Number of children	Percent (%)
Children aged 0–23 months		
Gender		
Female	2,112	48.8
Male	2,215	51.2
Age		
0–5	1,048	24.2
6–11	1,042	24.1
12–17	1,161	26.8
18–23	1,076	24.9
H ad diarrhea in the past two weeks		
No	3,416	82.4
Yes	728	17.6
Average birth weight		
Low than average	185	6.6
Average & higher	2,630	93.4
Vitamin A		
No	1,773	56.6
Yes	1,360	43.4
Deworming		
No	1,320	61.9
Yes	814	38.1
Mother characteristics		
Age		
15 – 24	1,735	40.1
25 – 34	1,938	44.8
35 – 49	655	15.1
BMI		
Lower than 18.5	312	7.3
Normal BMI (18.5 – 24.9)	3,020	70.3
Overweight (25.0 – 29.9)	641	14.9
Obesity (higher than 29.9)	320	7.4
Education		
No Formal education	836	19.3
Primary education	2,761	63.8
Secondary and higher education	730	16.9
Household characteristics		
Residence type		
Rural	3,117	72
Urban	1,210	28
Source of water		
Improved	2,411	55.7
Not improve	1,916	44.3
Time to take water		
Less than 30 min	2,038	46.9
More than 30 min	2,299	53.1
Type of toilet		
Unimproved	3,210	74.2
Improved	1,117	25.8

### 3.2 Infants feeding practices

The result from Table 2 shows more than half (56.6%) of children under 6 months were exclusively breastfed. Also, the majority of children (86.5%) continued breastfeeding up to 1 year. However, only 36.9% were breastfed up to 2 years. Furthermore, the majority of children aged 6–23 months, 75.6% and 63.55%, did not receive minimum dietary diversity and minimum meal frequency, respectively.

**TABLE 2:** Description of infants feeding practices

Variables	Number	Percent (%)
Exclusive breastfeeding (EBF) for under 6 months		
No	454	43.4
Yes	594	56.6
Exclusive Breastfeeding (EBF) at 1 year (12–15 months)		
No	103	13.5
Yes	661	86.5
Continue Breastfeeding (CBF) at 2 years (20–23 months)		
No	454	63.1
Yes	266	36.9
A child with Minimum Dietary Diversity (MDD), 4 of 7 food groups 6–23		
No	2,480	75.6
Yes	799	24.4
Children with Minimum Meal Frequency (MMF) for 6–23 months		
No	2,084	63.5
Yes	1,195	36.5

### 3.3 Bivariate and multivariate analysis of risk factors associated with Underweight

Multicollinearity test found there was a moderate correlation between independent variables so they all included in regression model. Backward multivariate regression process with 6th step was done revealing six variables significant predicts underweight: child's gender, age, birth weight, mothers' BMI, and education level as well as toilet facility (Table 3).

**TABLE 3:** Results for logistic regression analysis predicting the likelihood of a child to be underweight

Variables	Frequency (*N*)	Percent (%)	Crude OR (95% CI)	*p*‐value	Adjusted OR (95% CI)	*p*‐value
Gender						
Female	201	10.1	0.67 (0.55–0.81)	<.001	0.62 (0.48–0.81)	<.001
Male	296	14.3	1		1	
Age						
0–5	67	6.7	0.36 (0.26–0.48)	<.001	0.32 (0.21–0.48)	<.001
6–11	115	11.7	0.66 (0.51–0.85)	.002	0.68 (0.48–0.96)	.029
12–17	151	13.8	0.8 (0.63–1.019)	.071	0.84 (0.61–1.16)	.288
18–23	163	16.8	1		1	
Diarrhea						
No	389	11.7	0.75 (0.6–0.94)	.014		
Yes	108	15				
Birth weight						
Lower than average	35	22.9	2.74 (1.84–4.09)	<.001	2.92 (1.92–4.43)	<.001
Average & higher than average	242	9.7	1			
Vitamin A						
No	272	15.7	1.38 (1.12–1.7)	.003		
Yes	157	11.9				
Deworming						
No	218	17.1	1.49 (1.15–1.93)	.003		
Yes	96	12.2				
Mother age						
15–24	196	12.1	0.74 (0.57–0.96)	.022		
25–34	204	11.2	0.87 (0.52–0.88)	.003		
35–49	97	15.8	1			
BMI						
Lower than 18.5	54	17.9	2.97 (1.73–5.08)	<.001	2.48 (1.34–4.58)	.04
Normal (18.5–24.9)	378	13.2	2.08 (1.31–3.31)	.002	1.42 (0.86–2.36)	.17
Overweight (25.0 – 29.9)	43	7.3	1.8 (0.58–1.87)	.773	0.71 (0.38–1.33)	.262
Obesity (<=30)	20	6.7	1		1	
Education level						
No education	113	14.2	2.22 (1.56–3.17)	<.001	1.67 (1.02–2.73)	.04
Primary education	335	13.1	2.02 (1.47–2.77)	<.001	1.78 (1.23–2.58)	.002
Secondary and higher education	48	7	1		1	
Residence						
Rural	410	13.9	1.9 (1.49–2.43)	<.001		
Urban	86	7.8	1			
Source of water						
Unimproved	298	13.1	1.2 (1–1.46)	.055		
Improved	199	11.1	1			
Time to the water source						
>30 min	245	12.8	1.1 (0.92–1.54)	.298		
≤30 min	251	11.7				
Type of toilet facility						
Unimproved	410	13.6	1.7 (1.34–2.17)	<.001	1.44 (1.06–1.95)	.021
Improved	87	8.4	1		1	
Feeding practices						
EBF						
No	30	7.2	1.14 (0.69–1.87)	.618		
Yes	37	6.3	1			
Continue BF for 1 YR						
No	13	18.8	1.76 (0.92–3.38)	.087		
Yes	75	11.5	1			
Continue BF for 2 YR						
No	56	14.1	0.46 (0.31–0.69)	<.001		
Yes	69	26.2	1			
Child with MDD						
No	332	14.7	1.22 (0.96–1.55)	.11		
Yes	98	12.3	1			
Child with MMF						
No	270	14.5	1.08 (0.88–1.34)	.465		
Yes	160	13.5	1			

The odds of being underweight were lower in female children compared with male children (AOR = 0.62, 95% CI = 0.48–0.81) at, *p* < .001. The risk of being underweight was also less likely in children with three lower age groups: 0–5, 6–11, and 12–17 compared with the ages group of 18–23 months at *p* < .001. Similarly, the risk of being underweight was likely higher in children born with lower birth weight (AOR = 2.92, 95% CI = 1.92–4.43) compared to children born with an average or higher weight at *p* < .001.

Also, children born with mothers with BMI lower than 18.5 were more likely to be underweight (AOR = 2.48, 95% CI = 1.34–4.58) compared with those born with mothers with a higher BMI at *p* = .04. Likewise, the probability of being underweight was higher in children born with mothers with no education (AOR = 1.67, 95% CI = 1.02–2.73, *p* = .04) or primary education (AOR = 1.78, 95% CI = 1.23–2.58, *p* = .002) compared to those born with mothers with secondary or higher education.

Moreover, children from households with unimproved toilets were more likely to be underweight (AOR = 1.44, 95% CI = 1.06–1.95, *p* = .021) compared to their counterparts.

## 4 DISCUSSION

Multivariate analysis found children's age, gender, birth weight, mother's level of education, and BMI, as well as the type of toilet facilities used in the households were all revealed to be significant predictors of underweight in this study.

The low likelihood of female children being underweight found in this study mirrors the findings described previously in Rwanda and South Africa (Lesiapeto et al., 2010; Mukabutera et al., 2016). Female children's reduced risk of being underweight may be due to their low calorie requirements, which can be easily met by typical feeding practices, such as when mothers assure on‐demand breastfeeding. In contrast, other studies conducted in Pakistan, India, Indonesia, Ghana, and Kenya indicated that female children were more likely to be underweight than male children (Kumar et al., 2019; Acquah et al., 2019; Sari et al., 2020 and Stalin et al., 2013).

Children in the age group of 18–23 months were significantly more likely to be underweight compared with other groups. This result is in line with a previous study conducted in Ghana, which indicated that children aged 12–23 months had a higher chance of being underweight than children of other age groups (Acquah et al., 2019). This could be attributed to increased physical activity at this age when children can walk and run, as well as a decrease in mothers' care for these children due to the assumption that they are already grown up. Therefore, more training and counseling for mothers/child caretakers on proper and consistent feeding of all children under the age of 2 years when they attend postnatal clinics is needed to guarantee that children acquire weight (0.5 kg per month) as suggested by WHO. However, this result was different from studies conducted in Uganda and Kenya which show children less than 12 months are more likely to be underweight and stunted compared to older groups (Habaasa, 2015).

Furthermore, significantly higher risks of underweight were found for children born with a lower birth weight compared to other children born with an average or higher than average weight. Similar results were reported in Indonesia (Sari et al., 2020), Kenya (Gewa & Yandell, 2012), Pakistan (Kumar et al., 2019), and Nepal (Adhikari et al., 2017). Low birth weight has been linked to a variety of health issues, including infant respiratory distress syndrome and neonatal deaths. To ensure proper weight gain throughout pregnancy and the delivery of healthy and appropriate‐weight babies, all pregnant women should receive counseling, which should include early attendance at prenatal clinics, good nutrition (dietary diversity), and regular intake of prenatal vitamins and minerals, such as folic acid and iron supplements.

Moreover, the likelihood of being underweight was higher for children who were born with mothers who had a BMI lower than 18.5. The significant correlation between underweight and the mother's BMI was also reported in the studies conducted in Ethiopia, Ghana, Rwanda, and Nepal (Acquah et al., 2019; Adhikari et al., 2017; Mukabutera et al., 2016; Yisak et al., 2015). The physiologic effects of maternal malnutrition during lactation probably have an impact on milk secretion and a child's weight gain. Therefore, interventions aimed at improving maternal nutrition may be useful in reducing childhood malnutrition.

Likewise, a mother's education emerged as a significant factor that could predict being underweight for children surveyed. The increased risk of being underweight for children belonging to mothers with low education was also reported in the studies conducted in Rwanda (Mukabutera et al., 2016), South Africa (Lesiapeto et al., 2010), and Pakistan (Kumar et al., 2019). Mothers with a low level of education may be missing out on some essential information about infant and young child feeding practices, resulting in poor feeding and weight gain for their children. Thus, nutrition education should be organized for mothers/care givers during postnatal visits, depending on their level of education, or giving priority to low‐educated mothers/care givers.

The likelihood of being underweight was also higher for children from households that used unimproved toilets when compared with those who used improved toilets. This was in line with the studies conducted in Rwanda (Mukabutera et al., 2016), Pakistan (Kumar et al., 2019), and South Africa (Modjadji & Madiba, 2019). Having unimproved toilets results in poor sanitation, which would make children more vulnerable to infections and illnesses that ultimately cause underweight. Government through its responsible ministries should sensitize citizens on the importance of construction and use of improved toilets in order to stop all preventable diseases related to poor sanitation and finally reduced the risks of underweight in children.

The utilization of a wide sample size, comprising people from all over the country, gave the study enough power and improved generalization. However, the cross‐sectional structure of the study made it impossible to establish a causal effect relationship between predators and the variable of interest (underweight). Recalling bias was also observed in this study when caregivers/parents were required to provide some information like feeding practices. It is advised that future large studies take into account the identified limitations.

## 5 CONCLUSION

The child's age, gender, birth weight, mother's level of education, and BMI, as well as the type of toilet facilities utilized in the households were all revealed to be significant predictors of underweight in this study. Specific nutrition and health interventions are encouraged to ensure recommended weight gain and prevent the underweight burden for this age group. As part of interventions, regular growth monitoring, nutrition counseling, and nutrition education are essential to all parents/caregivers with children under 5 years of age when visiting postnatal clinics.

## CONFLICT OF INTEREST

The authors declare no conflict of interest.

## ETHICS APPROVAL AND CONSENT TO PARTICIPATE

The permission to do this study was given by the demographic and health surveys (DHS) program.

## Data Availability

Data for this study are available upon request from the Demographic and Health Survey (DHS) portal (www.dhsprogram.com).
